# A General Neural Network Model for Complex Refractive Index Extraction of Low-Loss Materials in the Transmission-Mode THz-TDS

**DOI:** 10.3390/s22207877

**Published:** 2022-10-17

**Authors:** Zesen Zhou, Shanshan Jia, Lei Cao

**Affiliations:** State Key Laboratory of Advanced Electromagnetic Engineering and Technology, Huazhong University of Science and Technology, Wuhan 430074, China

**Keywords:** terahertz (THz) spectroscopy, optical parameters, extraction, neural network

## Abstract

The complex refractive index for low-loss materials is conventionally extracted by either approximate analytical formula or numerical iterative algorithm (such as Nelder-Mead and Newton-Raphson) based on the transmission-mode terahertz time domain spectroscopy (THz-TDS). A novel 4-layer neural network model is proposed to obtain optical parameters of low-loss materials with high accuracy in a wide range of parameters (frequency and thickness). Three materials (TPX, z-cut crystal quartz and 6H SiC) with different dispersions and thicknesses are used to validate the robustness of the general model. Without problems of proper initial values and non-convergence, the neural network method shows even smaller errors than the iterative algorithm. Once trained and tested, the proposed method owns both high accuracy and wide generality, which will find application in the multi-class object detection and high-precision characterization of THz materials.

## 1. Introduction

The material properties, such as refractive index or dielectric constant, are indirectly measured through light-matter interactions. From the aspect of optical experiment approach, the transmission-mode terahertz time domain spectroscopy (THz-TDS) [1] is a mature technique for the corresponding waveform and spectrum measurement. The THz-TDS method has apparent advantages of simultaneously obtained amplitude and phase spectrum after fast Fourier transform (FFT) operation on the measured transmitted time domain THz field, high signal-to-noise ratio (SNR) with lock-in amplifiers, easiness to analyze the spectrum, adaptability for measuring thin samples [2,3]. The transfer function is firstly measured for normally incident THz pulses on the parallel interfaces. Starting from the measured data containing both amplitude and phase of the THz signal, many reference requiring methods and algorithms were proposed to estimate the optical parameters and/or the thickness of homogeneous material with the minimum error [4,5,6,7,8,9,10]. Based on the Fresnel formula for materials under plane wave excitation, the simple analytical method produces approximate results of complex refractive index with fast speed, although the accuracy is low especially for the refractive index *n* of high-loss and high-dispersion materials [11,12]. The iterative algorithms have high accuracy if the initial values are properly assigned, such as Newton-Raphson [13] and Nelder-Mead [14]. However, the problems of non-convergence in numerical calculation and low-speed in processing a large amount of spectroscopic data become the major disadvantages, limiting its applications in real-time scenarios [15].

The neural network becomes a novel strategy to deal with regression and classification problems with high accuracy, since the well-trained networks can model a nonlinear function of arbitrary complexity [16,17]. In the THz-TDS experiment, the neural network can be used to establish a mapping relationship between the experimental data and optical parameters of materials under test, which inherently replaces the inverse function via approximate formula or numerical approach. When the trained network behaves both extremely small (similar) values of deviation in the training set and variance in the test set, the final predictions on the experimental data will become credible without the “over-fit” phenomenon [18]. The artificial neural network finds initial applications in extracting both the refractive index and thickness of single-layer optical thin films in the visible region [19,20,21]. With the improvement of computer performance and the rise of many new disciplines and technologies, the use of neural network method in material characterization extends to quasi-crystalline alloy (Al_80_Mn_20_) [22], silicon photonics [23], 2D materials (MoSe_2_, WS_2_, WSe_2_) [24], 3D nanonetwork silicon structures [25], and binary ionic liquid system [26,27]. Recently, a new standard was proposed to evaluate the reliability of the optical parameter measurement of thin films via the neural network method [28].

The utilization of artificial intelligence techniques in THz region mainly concerns material reflection and/or transmission spectrum [29], and mostly solves classification problems including tag identifications [30], recognition of cancers [31], identification of components [32], and so on. Universal machine learning, especially the multifunctional neural network model, has better performance than traditional modeling techniques. Nevertheless, only a few such supervised regression-type applications have been demonstrated in the refractive index extraction of THz materials to the best of our knowledge. A U-net structure neural network was constructed to extract the thickness, refractive index, and absorption coefficient of SiO_2_ thin film from the Fourier transform infrared spectroscopy (FTIR) measurement [33], but it is only suitable for a few semiconductor materials. The artificial neural network method was preliminarily proved to extract the complex refractive index from THz-TDS data with higher accuracy than analytical method [34]. However, a general and easy-to-implement neural network model is still needed in this area. We aim to design a general network model to treat various materials with a wide range of thickness in the frequency range covering most THz-TDS systems, which can make up for the shortage of existing extraction methods at THz frequency.

In this paper, we illustrate the principle and procedure of building a general neural network model for optical parameter extraction in the THz frequency range. In view of most applications, the ranges of refractive index *n* and extinction coefficient *k* of randomly simulated materials in the trained model are (1, 5) and (0, 0.1), respectively. The maximal material thickness is 5 mm and the frequency is selected up to 20 THz. Further extension of these parameters is possible at the expense of more training time. In Section 2, the complete process of designing, training, and optimizing the neural network is described in detail, and the 4-layer network is found to simultaneously meet the requirements of wide generality and high accuracy with relatively less time-consuming. In Section 3, two measured materials (TPX, z-cut crystal quartz) and one simulated material (6H SiC) of different thicknesses are used as typical examples to validate the accuracy of our proposed general neural network model. Comparisons with iterative algorithm method (Nelder-Mead) are also conducted to represent the pros of the novel method. Finally, the conclusion is given in Section 4.

## 2. Neural Network Method

**Figure 1:**
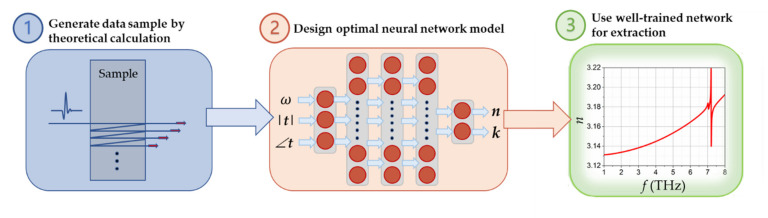
Flow chart of neural network method to extract optical parameters of materials from the transfer function.

Figure 1 describes the complete process to train and optimize the neural network for parameter extraction, based on the classical transfer function. The network is well trained to extract parameters point by point in the whole frequency range. Firstly, sufficient and reasonable data samples are generated randomly by theoretical calculation from the Fresnel formula [1]. To ensure the wide applicability of the model, the randomly generated training samples must cover the most possible kinds of materials. Secondly, the network is trained by repeatedly changing hyperparameters in order to find the best network configuration. A well-trained network should have small errors on both training set and test set, namely with good model generalization abilities. Meanwhile, the network with less training time to reach the expected loss goal is preferred. The model with relatively smaller errors and less time in the training process will be selected as the optimal one. Finally, the well-trained network is utilized to extract optical parameters in real time with the measured/simulated transmission spectrum. More details about the neural network method will be given in the following part.

### 2.1. Data Generation

The transfer function is theoretically calculated by [35],

(1) $$H\left(\omega \right)=\frac{4\left(n-jk\right)}{{\left(n-jk+1\right)}^{2}}\cdot e^{-j\left(n-jk-1\right)\omega d/c}\cdot FP\left(\omega \right),$$

(2) $$FP\left(\omega \right)={\left[1-{\left(\frac{n-jk-1}{n-jk+1}\right)}^{2}\cdot e^{-2j\left(n-jk\right)\frac{\omega d}{c}}\right]}^{-1},$$

where *ω* is the angular frequency, c is the light speed in vacuum, *d* is the material thickness and *FP* (*ω*) is the Fabry-Perot term. *n* and *k* represent the frequency-dependent refractive index and extinction coefficient of materials under test, respectively. If the material is optically thick, multiple transmitted pulses (echoes) from interface reflections can be well separated from the main transmitted pulse in the time domain signal. Therefore, the *FP* (*ω*) term in the transfer function in the frequency domain equals 1. While for optically thin materials, the echoes overlap with the main pulse in the time domain and therefore the complete form of *FP* (*ω*) should be considered.

Equation (1) serves as a bridge of transfer function between material optical parameters and transmission from the forward direction. In the THz-TDS system, the time domain signals of the reference and sample are measured firstly. After FFT operation and phase unwrapping, both the amplitude and phase of the transfer function are calculated. Therefore, the neural network *N*_1_ and *N*_2_ aims to build the expected relation from the backward direction,

(3) $$N_{1}\left(\tilde{t}\left(\omega \right),\omega \right)=n(\omega ),\;N_{2}\left(\tilde{t}\left(\omega \right),\omega \right)=k(\omega ),$$

The network model is trained with randomly simulated material parameters, frequency points and corresponding transfer function to well fit the backward relation in Equation (3). The mechanism for the accurate parameter extraction by neural network model relies on the internal relationship between material parameters and transmission spectroscopic values.

From Equation (3), the neural network consists of three input variables (angular frequency, amplitude, and phase of transmission at each frequency point) and two output variables (refractive index and extinction coefficient at each frequency point), which is also shown in Figure 1. The ∠t denotes the phase of the transfer function. The dataset is randomly generated beforehand by varying the three variables (*n*, *k*, and *ω*) in suitable ranges. For common materials in THz frequencies, the refractive index *n* is normally smaller than 5 and the extinction coefficient *k* for low-loss THz materials is normally hundreds of times smaller than *n* [34]. Therefore, the range of *n* and *k* in the dataset is chosen as (1, 5) and (0, 0.1) in the first step of data generation, respectively. The angular frequency range is 2π × (0.1, 20) THz, which covers the measured frequency range of most THz-TDS systems nowadays. The other required dependent variables ($\left|t\right|$ and ∠t) are theoretically calculated with Equation (1). For the number of data samples, an optimal value of 14,000 data samples (10,000 for training, 2000 for validation, and 2000 for the final test) is adopted in this regression task after numerous trials. If more data samples are used, the training time will dramatically increase while the loss level cannot be reduced further.

The thickness of most optically thick materials can be measured with high accuracy if a digital micrometer is used, and the thickness value is kept constant in the process of neural network training and prediction throughout this paper.

### 2.2. Neural Network Design

Deep neural networks can fit functions with high complexity due to the strong gradient descent algorithm, which is used in optimizing the internal weights to achieve very high precision. The extraction of optical parameters from the transmission spectroscopy data is essentially considered to be a fitting of Equation(3), and therefore the basic fully connected neural network is the best choice, which is believed to own better performance on normal regression tasks than other specific functional deep networks, like recurrent neural network (RNN) in natural language processing (NLP) [18], convolutional neural network (CNN) and generative adversarial network (GAN) in image processing [36,37]. Generally speaking, the optimal neural network model should basically meet the requirements of wide generality. In other words, the model must be applicable for common materials with different parameters (thickness, dispersion, dielectric constant, and losses) measured with most types of THz-TDS systems (operation frequency).

**Figure 2:**
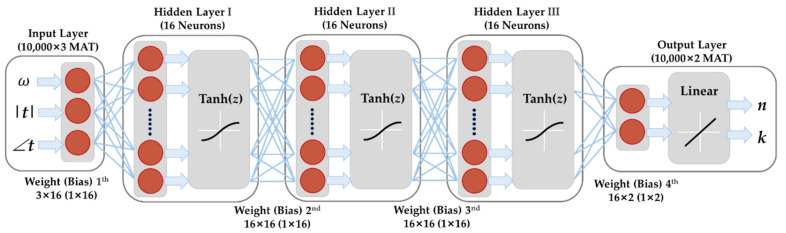
Schematic diagram of the optimal 4-layer neural network model.

The schematic diagram of the neural network model is shown in Figure 2, with three input variables in the input layer (layer 0), two output variables in the output layers (layer 4), and three fully connected hidden layers (each layer has 16 neurons). The input layer is not counted in the total number of layers of neural network. The total input of the network is a 10,000 × 3 matrix, and the output is a 10,000 × 2 matrix. To eliminate the dimension inconformity and obtain faster convergence, each variable in the generated data samples should be normalized beforehand. The total five variables are normalized between 0 and 1 with the linear normalization method, namely *x* * = (*x* − *x*_min_)/(*x*_max_ − *x*_min_). In the structure optimization process of neural network, only the number of layers and neurons is changed, since it has the greatest impact on network performance while other hyperparameters are kept the same. For the choice of activation functions, *Tanh*(*z*) = (e*^z^* − e^−*z*^)/(e*^z^* + e^−*z*^) is utilized in hidden layers because it is well suitable for the central symmetry problem and owns fast convergence speed in the training process. The neural network is trained with Levenberg-Marquardt (LM) algorithm, which behaves well in fitting a nonlinear function. LM is a second-order optimization algorithm which has faster and better convergence for neural network models than the basic first-order back propagation (BP) algorithm. All the programs are written and executed with the internally installed deep learning toolbox 14.4 in MATLAB R2022a.

The choice of the number of layers and neurons is critical in the design of neural network. The predefined validation sets are used to evaluate the performance of each network. As the simplest case, the 2-layer neural network with a single hidden layer can theoretically fit any continuous mapping relation in a short time [16], but the prediction accuracy still needs further improvement. For instance, if the number of neurons increases from 16 to 128, the error of *n* decreases dramatically while the error of *k* sustains high, due to the lack of network complexity. This negative effect could be alleviated by using the 3-layer neural network with two hidden layers. After a large amount of test with different combinations of neurons (8, 16, 32, 64, and 128) in the two hidden layers, the prediction accuracy could only be obtained for a few cases with specific combinations of neuron numbers (such as 128 × 8, 32 × 16 and 64 × 16), where the magnitude of deviation from the real values of *n* and *k* reaches the level of 10^−4^ and 10^−5^ at most frequency points, respectively. However, the time consumed in the training of the 3-layer network is constantly more than 10 h, which limits its practical applications in parameter extraction. Finally, the 4-layer neural network with three hidden layers (16 × 16 × 16 neurons) is selected, as shown in Figure 2. It possesses both higher accuracy and less training time. The evaluation of the optimal 4-layer network model will be discussed in the following part for a specific simulated material. In our work, we only tried several combinations of certain specific neuron numbers and satisfying results are produced in consideration of both accuracy and time. This could also be realized by the hyperparameter optimization framework (Optuna) at the sacrifice of much more time consumed [38].

### 2.3. Parameter Extraction

To quantitatively evaluate the accuracy of extracted results, appropriate parameters of statistical error are defined and utilized. If the real values of *n* and *k* are known (simulated materials SiC), the mean square error (*MSE*) and mean absolute percentage error (*MAPE*) are frequently used parameters to quantitatively evaluate the accuracy of the neural network, such that

(4) $$MSE=\frac{1}{N}\sum_{i=1}^{N}{\left(Y_{i}-{\hat{Y}}_{i}\right)}^{2},$$

(5) $$MAPE=\frac{100\%}{N}\sum_{i=1}^{N}\left|\frac{Y_{i}-{\hat{Y}}_{i}}{Y_{i}}\right|,$$

where *N* is the total number of frequency points, and *i* is the index of a specific frequency point. Variables with and without hat symbols are the corresponding extracted and real values. However, for the parameter extraction from actual THz-TDS data, the real values are unavailable (measured materials TPX and quartz) and the mean absolute error (*MAE*) of transfer function is utilized to compare the accuracy in an indirect way, between the neural network model and the iterative algorithm. It is defined as,

(6) $$MAE=\frac{1}{N}\sum_{i=1}^{N}\left|\left|t_{i}\right|-\left|{\hat{t}}_{i}\right|\right|+\frac{1}{2\pi }\left|\left|∠t_{i}\right|-\left|∠{\hat{t}}_{i}\right|\right|,$$

**Figure 3:**
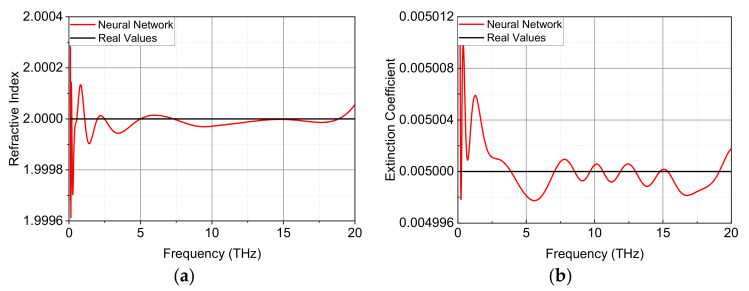
Comparison of extracted optical constants from neural network method and real values for a simulated non-dispersive material (*n* = 2 and *k* = 0.005) with a thickness of 3 mm: (**a**) Refractive index *n*; and (**b**) Extinction coefficient *k.*

An ideal non-dispersive thick material is used as the simulated material (test set) to preliminary test the actual extraction performance of the proposed 4-layetr network (16 × 16 × 16 neurons). Its thickness, refractive index and extinction coefficient are taken in the simulation as 3 mm, 2 and 0.005, respectively. The refractive index and extinction coefficient extracted with the neural network method are compared with the real values up to 20 THz, as shown in Figure 3a and Figure 3b, respectively. The magnitude level of deviation for *n* (*k*) reaches as low as 10^−5^ (10^−6^), which indicates the further improvement of accuracy over the case of 3-layer network. The average time needed for training such a 4-layer network is less than 2 h, which is much less than that for the 3-layer network. The time consumed in the prediction process is much less than that in the training process. For instance, the prediction time is 24.75 ms for 100 frequency points, 27.63 ms for 1000 frequency points and 27.96 ms for 10,000 frequency points. Obviously, the prediction time is not proportional to the number of frequency points, which is also the attractive nature of the neural network method. It should be noted all the programs run on a PC with CPU Intel Core i7-12700 and 32 GB RAM.

The thickness of sample is indeed an important parameter in the whole extraction process. In our proposed model, if the thickness of the material under test varies, the neural network should be re-trained with the new thickness. The dependency of the training time and the accuracy level on the material thickness has also been quantitatively calculated. Table 1 shows the values of *MSE* and *MAPE* of *n* and *k* for the same simulated material with varying thicknesses from 1 to 5 mm. Both the maximum values of *MSE* (5.91 × 10^−9^ for *n* and 3.93 × 10^−11^ for *k*) and *MAPE* (0.00225% for *n* and 0.191% for *k*) indicate that the 4-layer neural network has even higher accuracy than the iterative algorithm [35]. With regard to the training time, Table 1 also lists the averaged time consumption of the 4-layer network for material with different thicknesses. Besides, the training time of an individual 4-layer network reduces more than three times in comparison with the 3-layer network (>10 h).

It must be pointed out that the proposed neural network method can also deal with the case of taking the material thickness as an additional input variable. For example, the frequency range is chosen between 0.5 and 5.5 THz, and both *n* and *k* are constant as shown in Figure 3. Table 2 lists values of *MSE* and *MAPE* for extracted *n* and *k* with the neural network method, where different ranges of sample thickness are considered. In comparison with the case of fixed thickness, the accuracy of both *n* and *k* for the case of variable thickness becomes lower, in particular for material thickness in large ranges. Therefore, the neural network method with fixed material thickness is highly recommended for occasions of wide frequency range and high generality and accuracy.

Finally, if the sample thickness is expected as an output parameter besides the optical constants based on the transfer function, the present neural network model will need further modification because it only considers the principle transmitted pulse in the measured transmission spectrum. A possible solution to predict the unknown thickness is to include the 1st echo signal of the transmission spectrum in the neural network model. This method has been successfully tested for ideally simulated materials, and the feasibility and robustness need experimental verification in our future work.

In the next Section, we will further demonstrate the network performance with three specific materials with different levels of dispersion and different thicknesses to prove the generality.

## 3. Application Examples

The generality and robustness of the 4-layer neural network method is investigated in the optical parameter extraction of three types of low-loss actual materials (TPX, z-cut crystal quartz and 6H SiC) from the transfer function. For TPX with low-dispersion and z-cut crystal quartz with moderate dispersion, the spectroscopic data were obtained by the transmission-mode THz-TDS measurement from 0.5 to 4.5 THz. We use the general model to extract the optical parameters, and the results were compared with those obtained from the homemade code based on the Nelder-Mead algorithm [35]. For SiC with strongly dispersive behavior in the THz region, the transmission spectrum was simulated up to 8 THz to account for the resonant properties in the dielectric function. Besides, the extracted results from simulated transfer function are obtained by both neural network method and Nelder-Mead algorithm. Moreover, we will discuss the case of optically thin SiC material (0.05 mm) considering Fabry-Perot effect in the transmitted signal. In this situation, a specific deep neural network method was adopted by dividing the wide frequency range into small segments with equal length for the purpose of better training and prediction.

### 3.1. TPX and Quartz with THz-TDS Measurement

In the TDS measurement system (Advantest TAS7500TS), two optical fiber lasers (pulse duration 50 fs, center wavelength 1.55 μm, repetition frequency 50 MHz, average power 20 mW) were used in both THz generation and detection by photoconductive antenna [39]. The measurement is carried out in the dry air environment and the speed without mechanical delay line is fast (8 ms per scan), with an average time of 10 s for each sample. The original time scan range is 130 ps and the time and frequency resolution of the generated signal is 2 fs and 7.6 GHz respectively.

**Figure 4:**
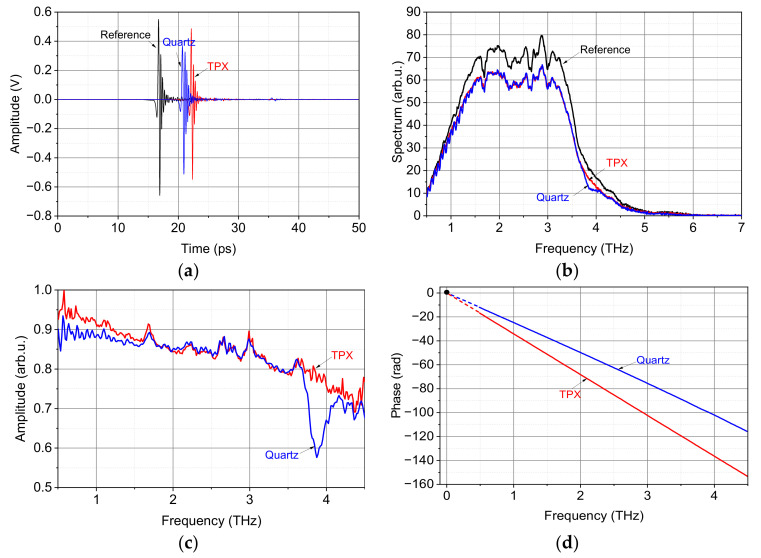
(**a**) Measured time domain signals of reference (black), TPX (red) and quartz (blue); (**b**) normalized transmission spectrum of reference (black), TPX (red) and quartz (blue); (**c**) amplitude of the transfer function of the two materials: TPX (red) and quartz (blue); (**d**) phase of the transfer function of the two materials: TPX (red) and quartz (blue).

Since the presence of multiples in the time domain signal will introduce echo oscillations in the spectrum [40] and their amplitudes are comparable to the noise floor, the original measured signal is truncated just before the appearance of the first multiple with suitable time window, as shown in Figure 4a. After FFT operation, Figure 4b shows the frequency domain spectrum of the transmitted pulse without (reference) and with object materials. In the reference measurement without sample, the SNR value becomes much lower for frequencies below 0.5 THz and beyond 4.5 THz in the transmission spectrum, and hence the effective frequency range is selected between 0.5 to 4.5 THz with high amplitude of THz signal, where the optical parameters are extracted later. By making the ratio between the sample spectrum and the reference spectrum, the magnitude and phase of the transfer function are easily calculated, which is shown in Figure 4c,d. The magnitude for both the two samples decreases with the increase of frequency. There is an absorption dip at 3.88 THz for crystal quartz, which is related to the lowest energy optical phonon mode [40]. As shown in Figure 4d, the slope of the phase curve for TPX is higher than that for quartz, because the TPX material is much thicker.

**Figure 5:**
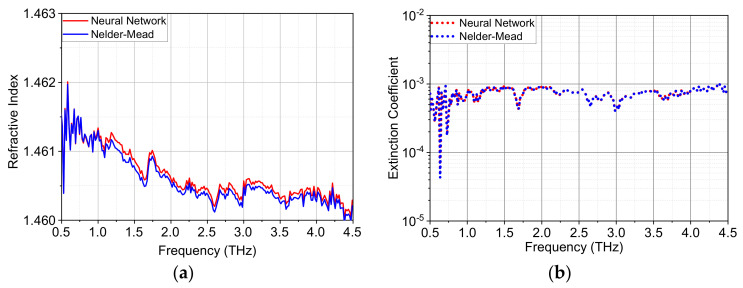
Comparison of extracted optical constants between neural network method and Nelder-Mead algorithm for TPX with a thickness of 3.538 mm: (**a**) refractive index and (**b**) extinction coefficient.

Figure 5 shows the extracted complex refractive index of TPX from 0.5 to 4.5 THz with both the neural network method and Nelder-Mead algorithm. The refractive index of TPX decreases slightly from 1.4613 at 1 THz to 1.4603 at 4 THz, which agrees with existing publications [41,42]. Good agreement between the two approaches have been achieved especially for the extinction coefficient *k* (Figure 5b), and the difference of *n* between them (Figure 5a) is in the order of 0.0001 or less. The value of *MAE* for the two materials is listed in Table 2. For TPX, the error is slightly reduced with the neural network method in comparison with the iterative algorithm.

**Figure 6:**
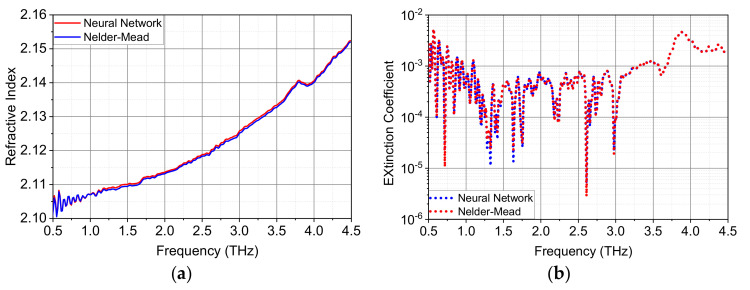
Comparison of extracted optical constants between neural network method and Nelder-Mead algorithm for quartz with a thickness of 1.068 mm: (**a**) refractive index and (**b**) extinction coefficient.

The extracted optical parameters for z-cut quartz with neural network and Nelder-Mead methods are shown in Figure 6. As for quartz, the refractive index increases from 2.107 at 1 THz to 2.152 at 4.5 THz, which shows a moderate frequency dispersion. The derivative-like feature in the refractive index and the corresponding peak in the extinction coefficient at around 3.8 THz is caused by the optical phonon mode, which is more obvious at low temperatures [40], and the absorption in quartz is higher than that in TPX material, in particular at high frequencies. Again, the results obtained from the two methods agree quite well. The MAE value for the dispersive quartz (Table 3) is reduced more than 8 times with the application of the neural network method.

The thickness value (3.538 mm for TPX and 1.068 mm for quartz) is kept as constant in each network configuration. If the thickness increases or decreases by 10 μm, the maximal relative error of *n* is calculated to be about 0.1% for TPX and 0.5% for quartz. The thickness uncertainty caused by measurement tools (commercial digital micrometer) is usually within several microns. This small variation of thickness has a negligible effect on the extracted value of *k*. Generally, the estimation error caused by thickness is small enough to be ignored for optically thick materials.

### 3.2. 6H SiC with Simulated Transfer Function

6H SiC is a strongly dispersive material in the THz region, and its complex permittivity can be expressed by the muti-Lorentzian function as [43],

(7) $$\varepsilon \left(\omega \right)=\varepsilon_{\infty }+\sum_{k=1}^{3}\frac{W_{k}}{\omega_{0,k}^{2}-\omega^{2}-j\omega /\tau_{k}}$$

where the parameters are *ε*_∞_ = 6.625, *W_k_* = [0.52, 1.85, 71342] ps^−1^, *τ_k_* = [4, 12.4, 2.61] ps, and *ω*_0,*k*_ = [7.034, 7.203, 23.87] THz. The relation between complex refractive index and relative permittivity is *ε* = (*n* − j*k*)^2^. The transmission spectrum of 0.5 mm thick SiC is theoretically simulated under the normal incidence of THz radiation without any noise and used for parameter extraction.

**Figure 7:**
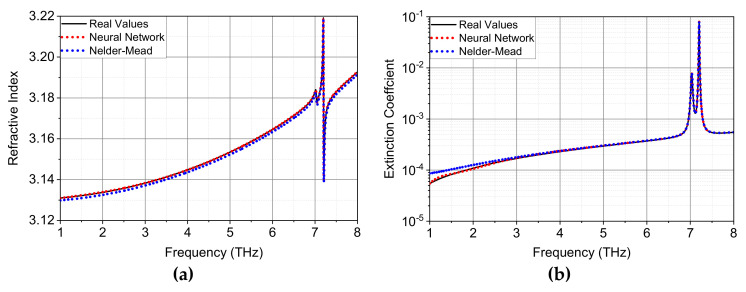
Comparison of extracted optical constants between real values and calculated results with neural network method and Nelder-Mead algorithm for optically thick SiC with a thickness of 0.5 mm: (**a**) refractive index and (**b**) extinction coefficient.

Table 4 summarizes the MSE and MAPE values of extracted *n* and *k* for the neural network method and Nelder-Mead algorithm. The errors with the neural network method are again extremely low, indicating a nearly perfect reproduction of real values. This is also intuitively validated by the extracted optical parameters up to 8 THz shown in Figure 7, where large resonances for *n* and *k* are observed at around 7 THz resulted from the strong dispersion of the dielectric function in Equation (7). In reference to the real values, it is evident that the extracted results with neural network method are much better than those with the Nelder-Mead algorithm in terms of accuracy, especially in the region where the extinction coefficient is extremely small.

From the above results, a conclusion could be drawn that the neural network method successfully extracts optical parameters of thick low-loss THz materials with extremely high accuracy. However, for optically thin materials (usually dozens of microns), the Fabry-Perot effect should be considered and therefore the transmitted signal will include multiple echoes. For an even wider versatility, the application of neural network method in the case of thin materials will be investigated in the following part.

**Figure 8:**
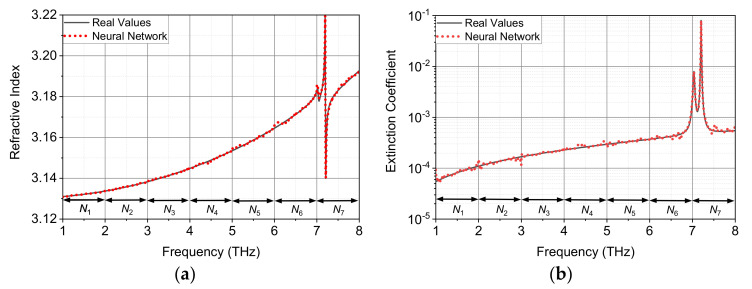
Comparison of extracted optical constants between the real values and neural network method for optically thin 6H SiC with a thickness of 0.05 mm: (**a**) refractive index and (**b**) extinction coefficient.

We still simulate the transmission spectrum of 6H SiC material with a thickness of 0.05 mm, where the FP (*ω*) term is taken as the full expression in Equation (2) in the data generation part. In this case, the relationship between input and output variables (Equation (3)) becomes complex and more sensitive to the minor change of variables, and therefore the original single neural network model for training and prediction should be revised accordingly to obtain high accuracy. After numerous tests, an effective way to overcome the above issue is to narrow down the range of independent variables (frequency, refractive index and extinction coefficient) for training. Among them, the frequency division is the easiest way. We divide the frequency range 1–8 THz at the interval of 1 THz and therefore a total of seven parallel neural networks (*N*_1_–*N*_7_) for predictions are needed. The network training and parameters extraction take place in the same frequency segment both in the training and extraction process. Figure 8 shows the extracted values independently calculated by the seven different networks, which agree well with the real optical parameters of SiC. The noise-like features in the curves of *n* and *k* can be ameliorated by using even smaller segment at the expense of slow extraction speed (more training time for more segment models). In order to reduce the training time for real applications, parallel computation seems to be a feasible solution at the expense of more hardware resources.

## 4. Conclusions

A general and efficient method based on 4-layer neural network has been proposed for the optical parameter extraction of low-loss materials based on the transmission-mode THz-TDS measurement. Three types of low-loss materials (TPX, z-cut quartz and 6H SiC) with different level of frequency dispersion characteristics and different thicknesses in the THz frequency range are used as typical examples to validate the robustness of the neural network. Good agreements between the neural network method and Nelder-Mead algorithm (TPX and z-cut quartz) or the real optical parameters (6H SiC) have been achieved. In comparison with the traditional iterative algorithm, the advantages of neural network method are the versatility for a wide range of materials with different thicknesses, non-necessity of initial values and even higher accuracy (lower error values). If the thickness is taken as an additional input variable for an unknown material, the maximal frequency and accuracy of the extracted results will become lower. The neural network method is expected to find applications in the multi-class object detection and high-precision characterization of low-loss materials in THz frequencies.

## Figures and Tables

**Table 1:** Training time, MSE and MAPE values of the 4-layer network for material with different thicknesses.

Thickness (mm)	Time Consumed (Hour, Minute)	*MSE*		*MAPE*	
		*n*	*k*	*n*	*k*
1	1, 56	3.59 × 10^−9^	3.93 × 10^−11^	0.00175%	0.0746%
2	1, 39	5.91 × 10^−9^	3.13 × 10^−11^	0.00225%	0.0900%
3	1, 41	4.37 × 10^−9^	3.35 × 10^−11^	0.00171%	0.0906%
4	1, 34	3.49 × 10^−9^	3.07 × 10^−11^	0.00178%	0.191%
5	1, 06	1.88 × 10^−9^	2.36 × 10^−11^	0.00146%	0.174%

**Table 2:** MSE and MAPE values for *n* and *k* with sample thickness as a variable with different ranges.

Thickness Range (mm)	*MSE*		*MAPE*	
	*n*	*k*	*n*	*k*
(0.1, 1)	5.59 × 10^−8^	7.68 × 10^−11^	0.010%	0.133%
(0.1, 2)	2.90 × 10^−7^	1.59 × 10^−9^	0.022%	0.677%
(0.1, 3)	6.89 × 10^−6^	2.70 × 10^−8^	0.106%	2.384%
(0.1, 4)	4.89 × 10^−5^	7.40 × 10^−8^	0.282%	4.950%
(0.1, 5)	6.33 × 10^−5^	1.94 × 10^−7^	0.362%	7.298%

**Table 3:** MAE for TPX and z-cut quartz with the Nelder-Mead algorithm and Neural network method.

	Nelder-Mead	Neural Network
TPX	0.0059628	0.0055075
Quartz	0.0083644	0.0009987

**Table 4:** Statistical error of the prediction values of 6H SiC with the Nelder-Mead algorithm and Neural network method.

	Nelder-Mead		Neural Network	
	*MSE*	*MAPE*	*MSE*	*MAPE*
*n*	1.5105 × 10^−6^	0.0388%	7.85 × 10^−9^	0.0022%
*k*	1.042 × 10^−10^	5.6504%	1.9974 × 10^−11^	1.7847%

## Data Availability

The simulation files/data used to support the findings of this study are available from the corresponding author upon request.
